# 

**Published:** 1843-06-10

**Authors:** 


### CONTENTS.

A Course of Clinical Lectures, delivered at the Hôtel Dieu, Paris, for the Session 1842–'43. By A. F. Chomel, M.D. Lecture 9, . . . . . . 121

Case of typhoid fever, (Dothinenteritis) in which the first sound of the heart was scarcely audible—Death—Autopsy—With remarks. By C. W. Pennock, M. D. . . . . . 123

*Bibliographical Notices.*

Dissertation on the Diseases of the Maxillary Sinus. Read before the American Society of Dental Surgeons, at their Third Annual Meeting, held in Boston, July 20, 1842. By Chapin A. Harris, M.D., D.D.S. Professor of Practical Dentistry in the Baltimore College of Dental Surgery, &c. &c. Philadelphia: Lea & Blanchard. 1843. 8vo. pp. 165, . . . . . . 125

Quarterly Summary of the Transactions of the College of Physicians of Philadelphia. February, March and April, 1843. 8vo. pp. 34, . . . . . 126

Editorial—Homœopathic Literature 126

Influence of Climate on Pulmonary Consumption, . . . . . . 127

*Retrospect of the Medical Sciences.*

On the Special Function of the Skin. By R. Willis, M. D. . . . . 128

Physiology and Pathology of the Blood 129

Anæmia of the Brain induced by Fatal Hemorrhage. By G. Burrows, M.D. 129

Strumous Peritonitis, . . . . . 131

Meningeal Apoplexy, . . . . . 132

Illegal Practice of Medicine in Paris, . 132

CONTENTS.

A Course of Clinical Lectures, delivered at the Hôtel Dieu, Paris, for the Session 1842–'43. By A. F. Chomel, M.D. Lecture 9, . . . . . . 121

Case of typhoid fever, (Dothinenteritis) in which the first sound of the heart was scarcely audible — Death — Autopsy—With remarks. By C. W. Pennock, M. D. . . . . . . 123

*Bibliographical Notices.*

Dissertation on the Diseases of the Maxillary Sinus. Read before the American Society of Dental Surgeons, at their Third Annual Meeting, held in Boston, July 20, 1842. By Chapin A. Harris, M.D., D.D.S. Professor of Practical Dentistry in the Baltimore College of Dental Surgery, &c. &c. Philadelphia: Lea & Blanchard. 1843. 8vo. pp. 165, . . . . . . 125

Quarterly Summary of the Transactions of the College of Physicians of Philadelphia. February, March and April, 1843. 8vo. pp. 34, . . . . . 126

EDITORIAL—HOMŒOPATHIC LITERATURE 126

Influence of Climate on Pulmonary Consumption, . . . . . . 127

*Retrospect of the Medical Sciences.*

On the Special Function of the Skin. By R. Willis, M. D. . . . . 128

Physiology and Pathology of the Blood 129

Anæmia of the Brain induced by Fatal Hemorrhage. By G. Burrows, M. D. 129

Strumous Peritonitis, . . . . . 131

Meningeal Apoplexy, . . . . . 132

Illegal Practice of Medicine in Paris, . 132

